# Supplementary material for: Indoleamine dioxygenase and tryptophan dioxygenase activities are regulated through control of cell heme allocation by nitric oxide
Source: J Biol Chem. 2023 Apr 26;299(6):104753. doi: 10.1016/j.jbc.2023.104753 (PMC10220489; doi:10.1016/j.jbc.2023.104753)

Indoleamine Dioxygenase and Tryptophan Dioxygenase Activities are Regulated through Control of Cell Heme Allocation by Nitric Oxide

Pranjal Biswas and Dennis J. Stuehr*

Number	Page number
Table S1	S-2
Fig. S1	S-3
Fig. S2	S-4
Fig. S3	S-5
Fig. S4	S-6
Fig. S5	S-7
Fig. S6	S-8
Fig. S7	S-9
Fig. S8	S-10
Fig. S9	S-11
Fig. S10	S-12
Fig. S11	S-13
Fig. S12	S-14
Fig. S13	S-15
Fig. S14	S-16

Table S1. Kyn production activities of cell supernatants. Cells expressing IDO1 or TDO were cultured with or without 5µM NOC-18 6h prior to lysis. The cell supernatants either did or did not receive heme at 3 or 6 µM at the point of assay. Supernatant activities were compared to estimate the percentage of heme saturation in the cell IDO1 or TDO at the time of cell lysis. Data are mean +/- s.d. of three replicates.

**Condition**	**Supernatant activity (nmol/min/mg)**	**Calculated % heme content**
empty vector	-0.17 ± 0.04	0
IDO1-FLAG	4.4 ± 0.02	18
IDO1-FLAG, 3 µM Heme	24.9 ± 0.1	99
IDO1-FLAG, 6 µM Heme	25.0 ± 0.1	100
IDO1-FLAG + NOC-18	24.5 ± 0.1	97
IDO1-FLAG + NOC-18, 3 µM Heme	25.1 ± 0.1	99
IDO1-FLAG + NOC-18, 6 µM Heme	25.2 ± 0.1	100
empty vector	-0.17 ± 0.02	0
TDO-FLAG	13.3 ± 0.1	33
TDO-FLAG, 3 µM Heme	40.5 ± 0.2	99
TDO-FLAG, 6 µM Heme	40.7 ± 0.3	100
TDO-FLAG + NOC-18	38.9 ± 0.2	95
TDO-FLAG + NOC-18, 3 µM Heme	40.7 ± 0.3	99
TDO-FLAG + NOC-18, 6 µM Heme	40.8 ± 0.3	100

Fig S1: Rate of NO release from NOC-18 under our experimental conditions.

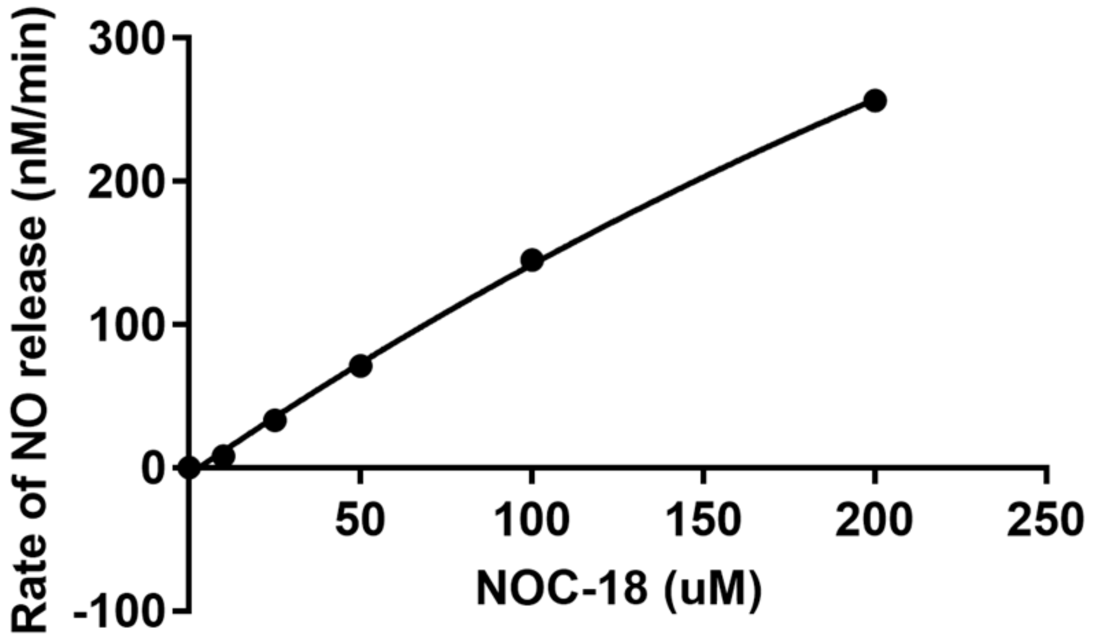

Fig S2: TDO expressions in different NOC-18 treated conditions in HepG2 cells. WB corresponds to Fig. 1.

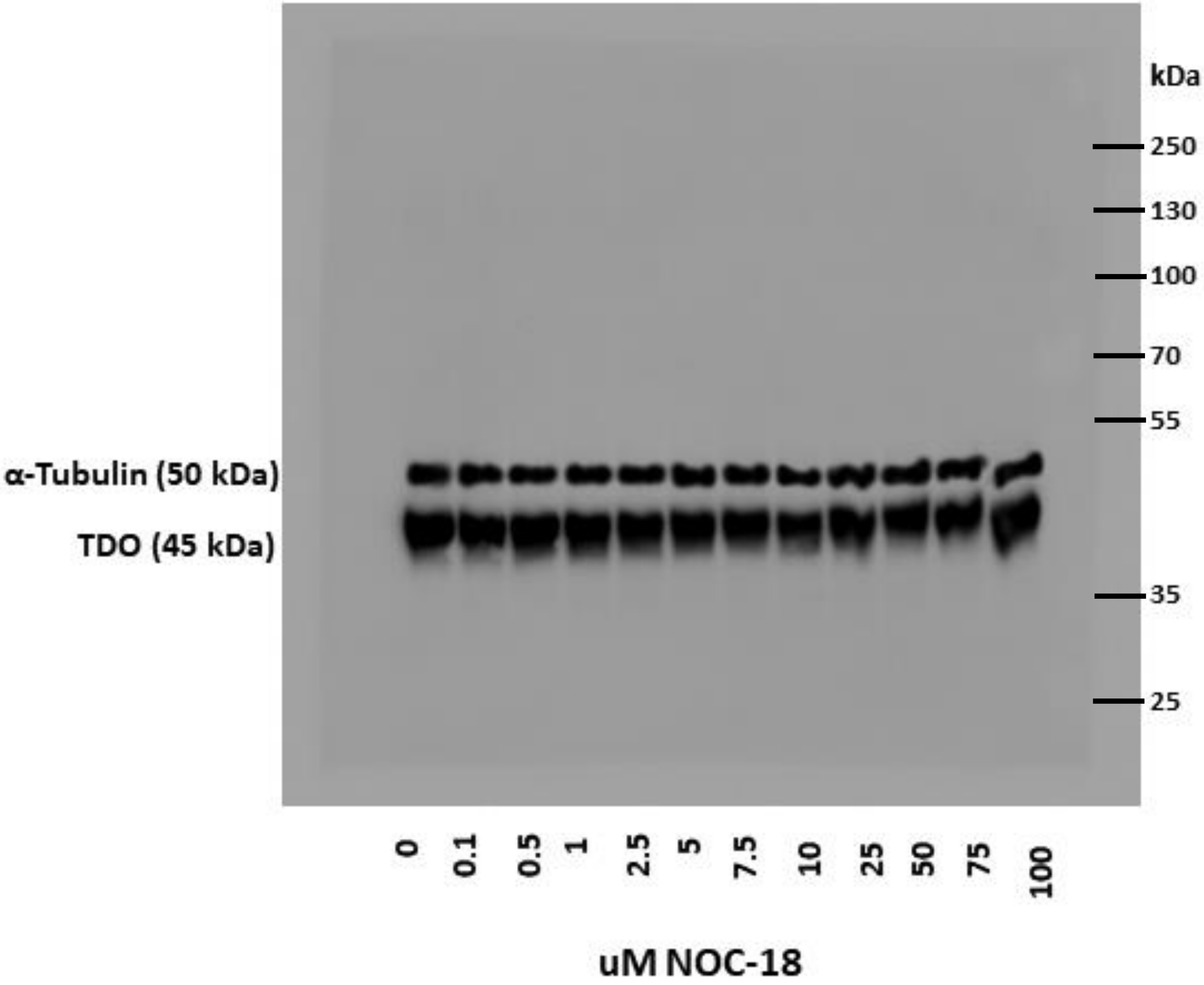

Fig S3: TDO-FLAG expressions in different NO donor treated conditions in HEK293T cells. WB corresponds to Fig. 1.

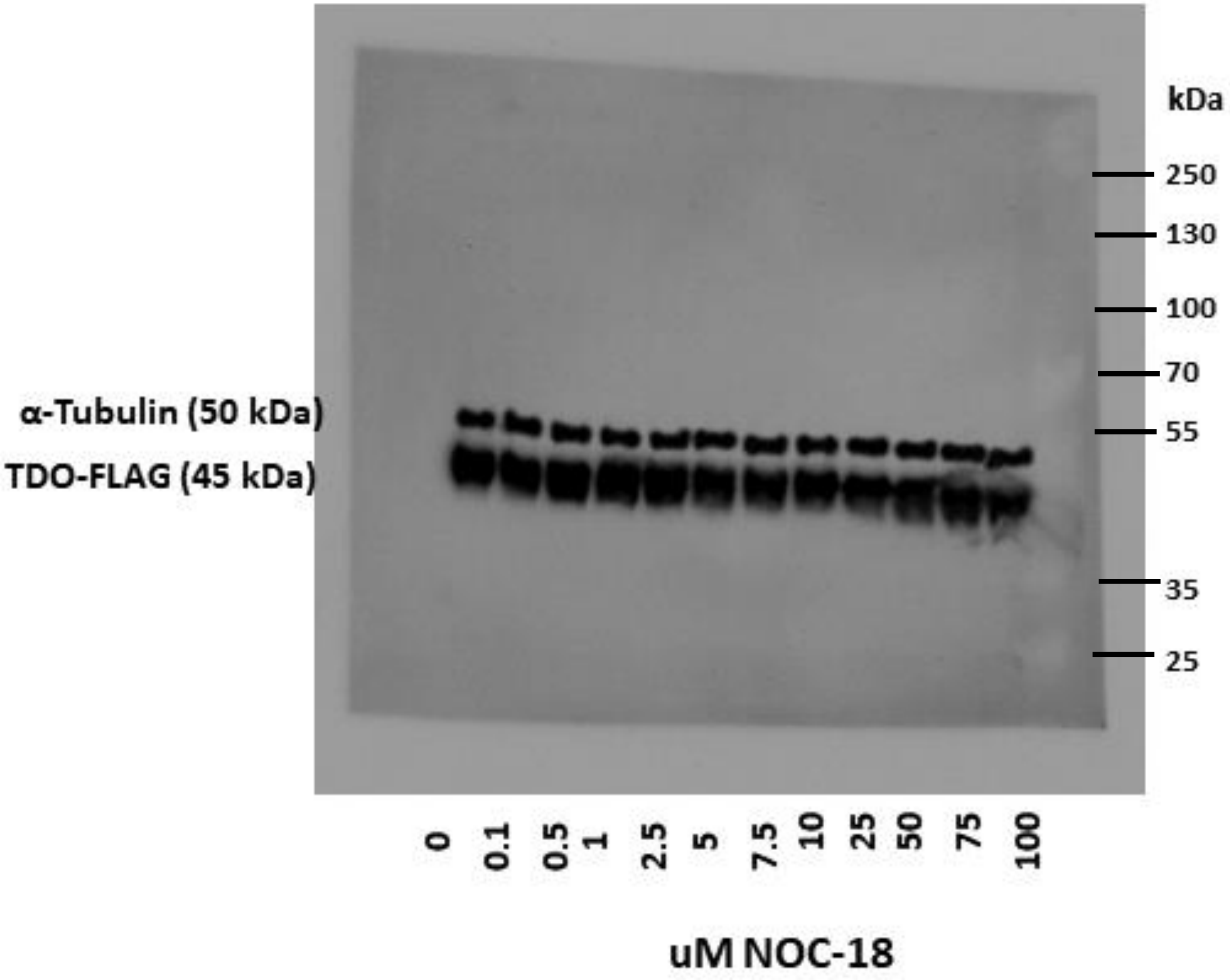

Fig S4: IDO1-FLAG expressions in different NOC-18 treated conditions in HEK293T cells. WB corresponds to Fig. 1.

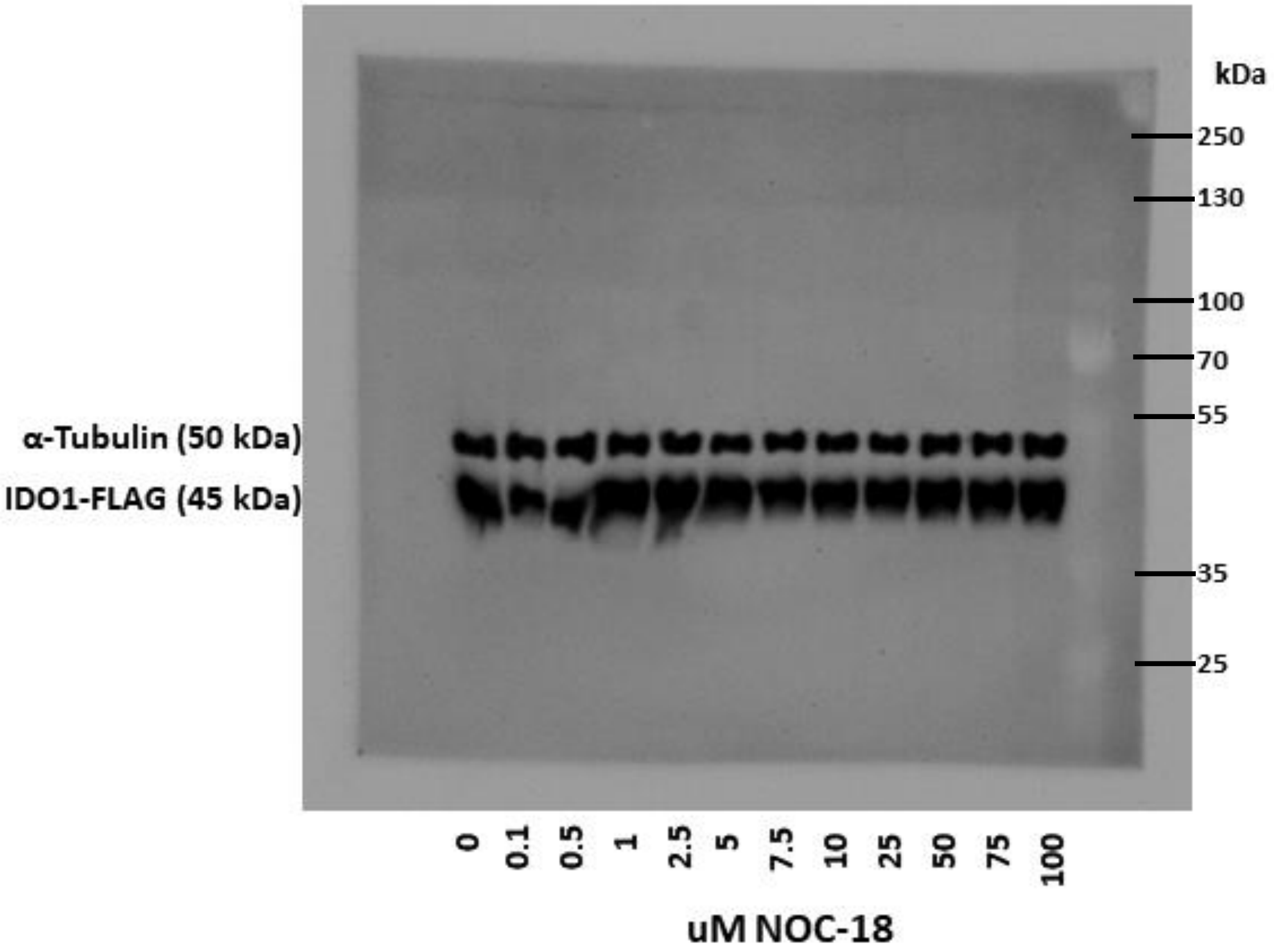

Fig S5: TDO-FLAG expressions in different NOC-18 treated conditions in GlyA-CHO cells. WB corresponds to Fig. 2.

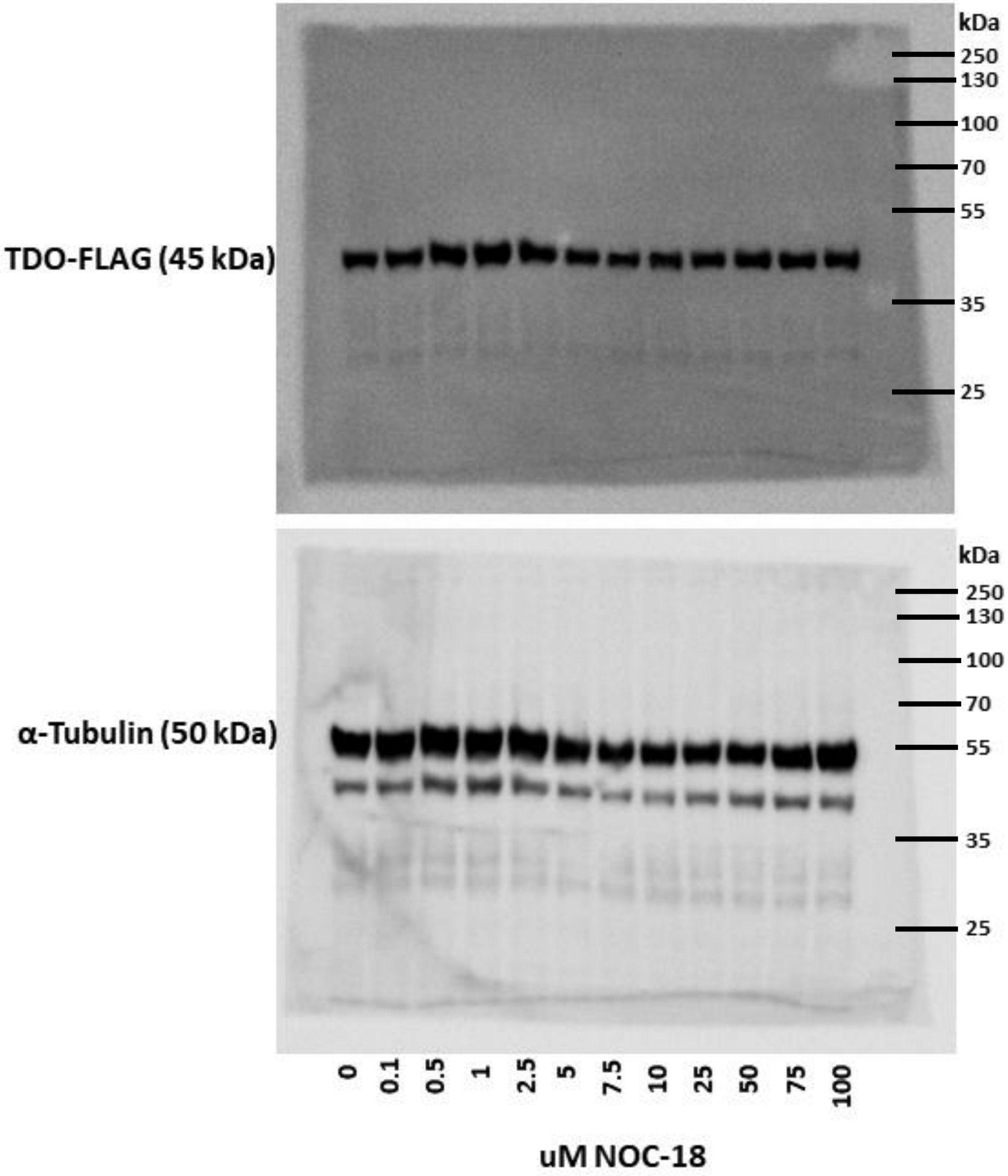

Fig S6: IDO1-FLAG expressions in different NOC-18 treated conditions in GlyA-CHO cells. WB corresponds to Fig. 2.

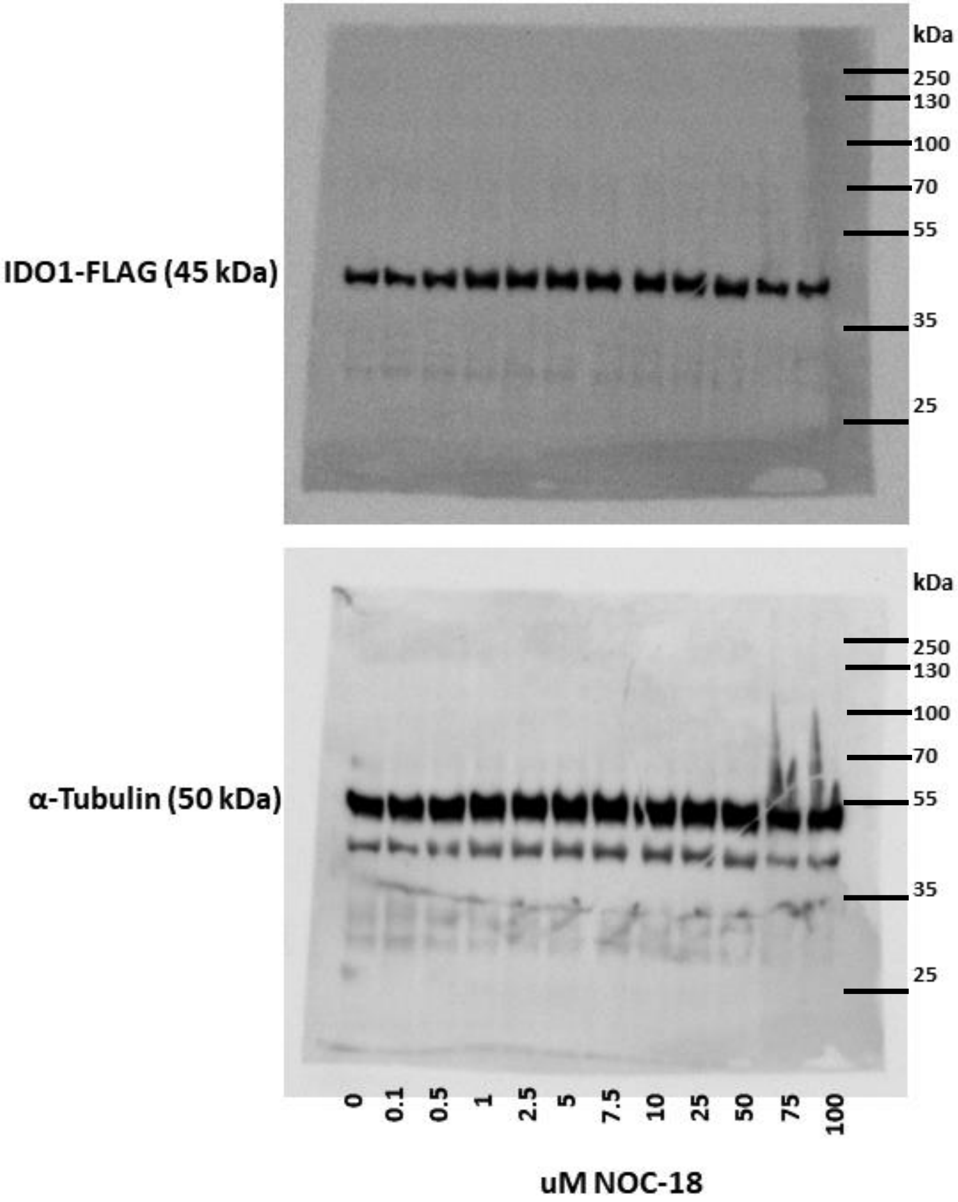

Fig S7: Expressions of IDO1-FLAG and TDO-FLAG in GlyA-CHO cells in the bottom chamber in the trans-well co-culture experiments with RAW264.7 cells in the upper chamber. RAW264.7 cells expression levels of iNOS upon activation with 1 µg/ml LPS for 6h. The WB corresponds to Figs. 3 and 4.

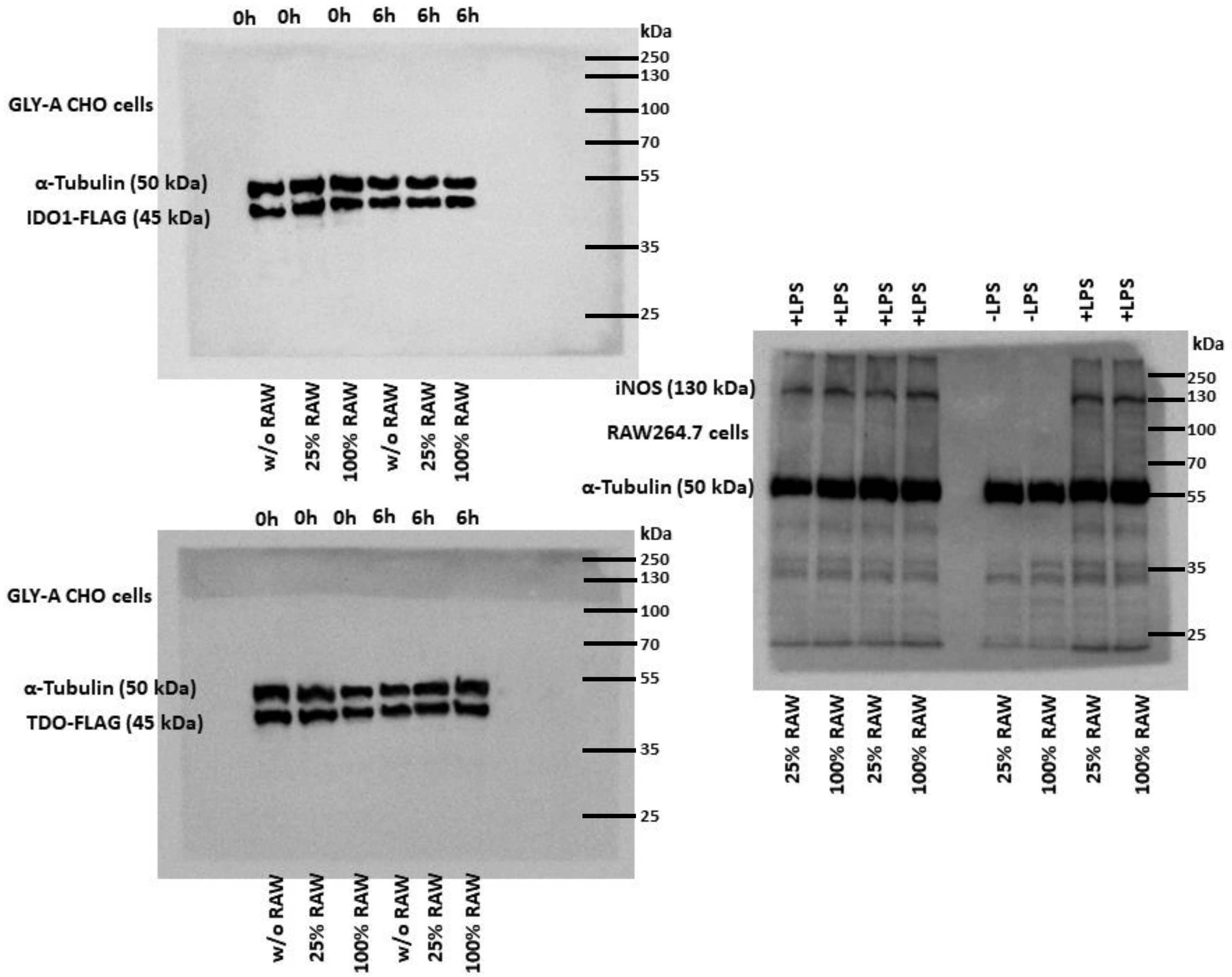

Fig S8: IDO1-FLAG expression levels in GlyA-CHO cells treated without, 5 µM and 100 µM NOC-18 for different time points as mentioned. WB corresponds to Fig. 5.

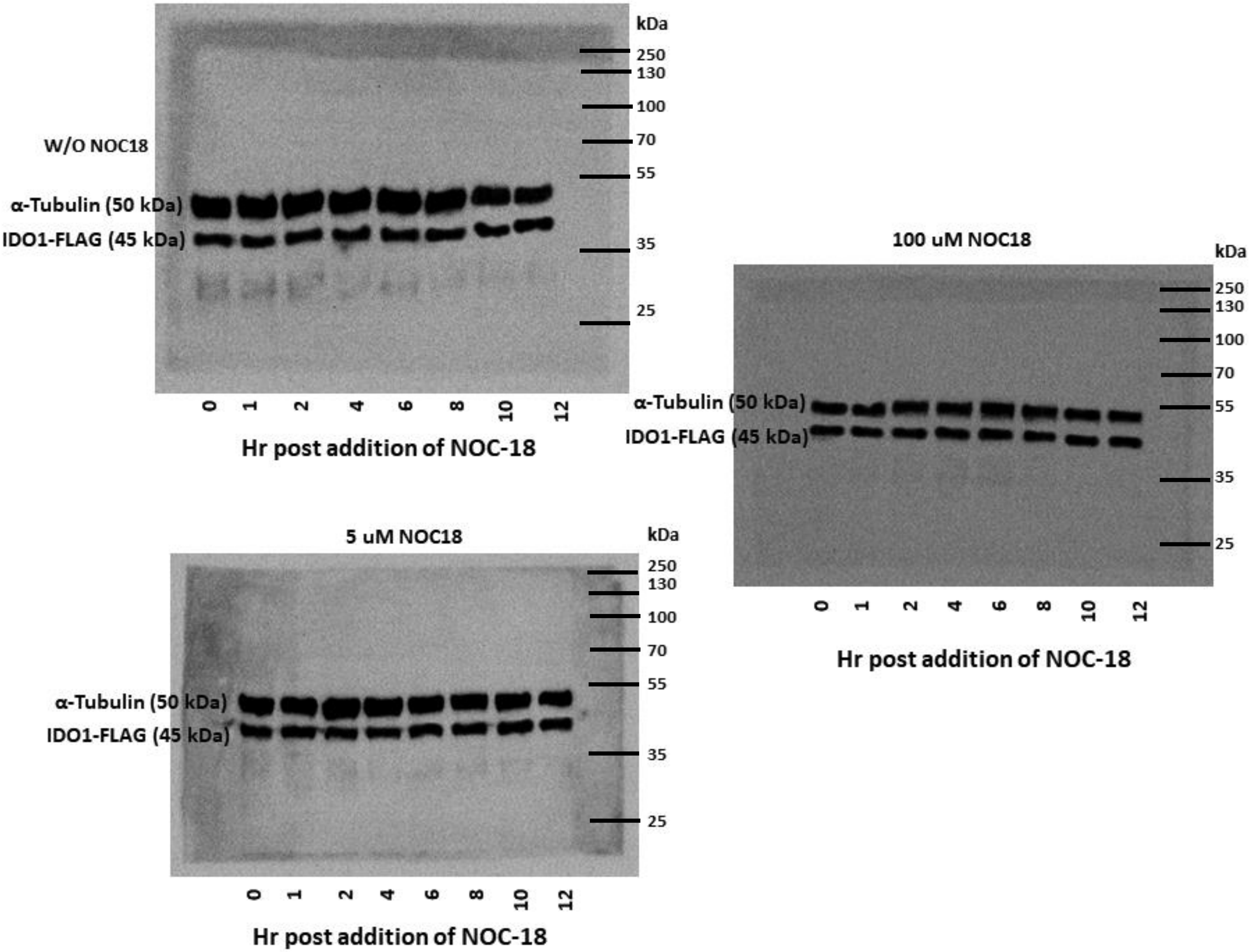

Fig S9: TDO-FLAG expressions in GlyA-CHO cells treated without, 5 μM and 100 μM NOC-18 for different time points as mentioned. WB corresponds to Fig. 5.

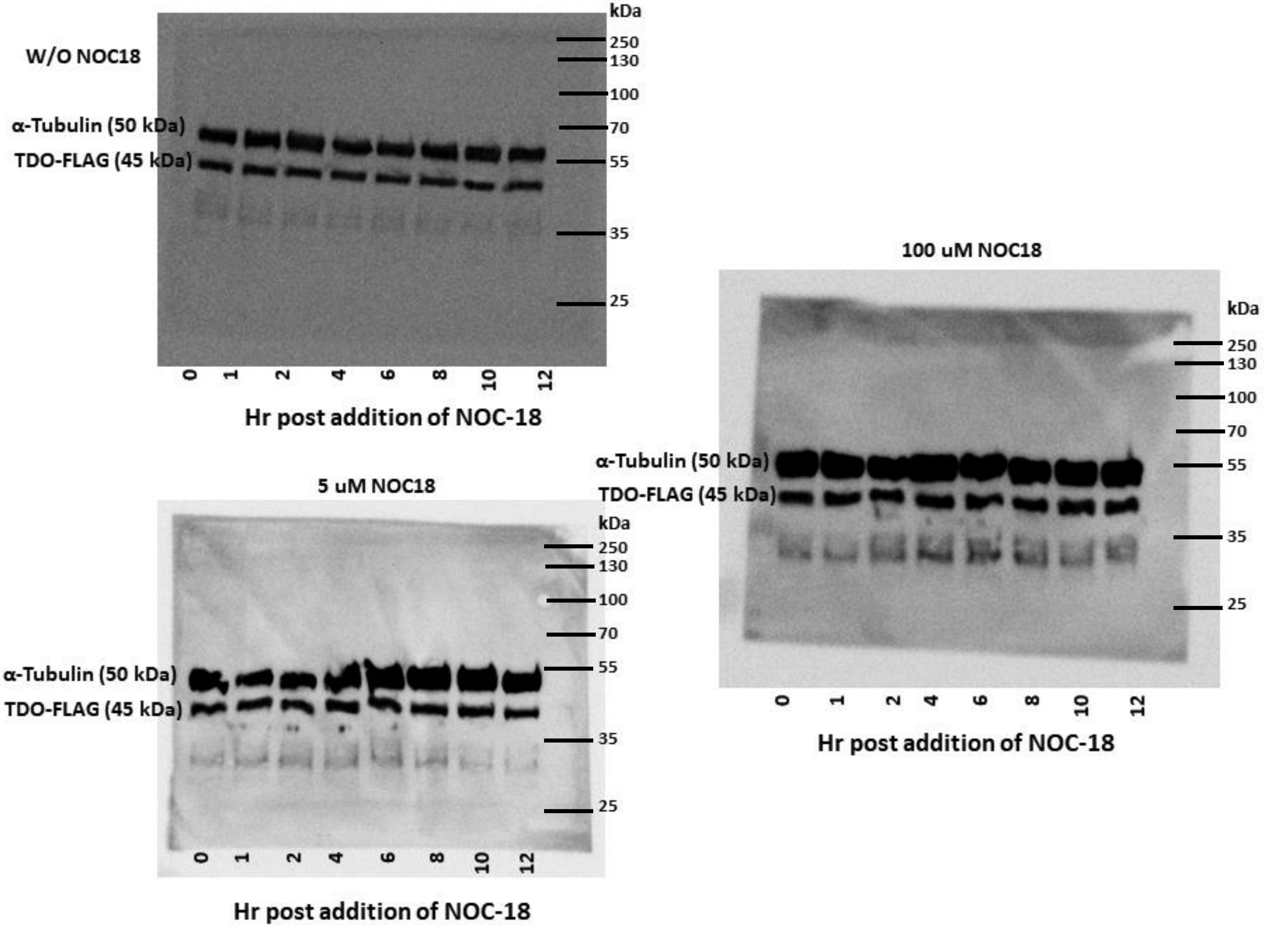

Fig S10: IDO1-FLAG expression in GlyA-CHO cells upon silencing of GAPDH with siRNA and complemented with either WT or H53A HA-GAPDH expression from transfected plasmid. GAPDH was effectively silenced as seen in the blot. WB corresponds to Fig. 6.

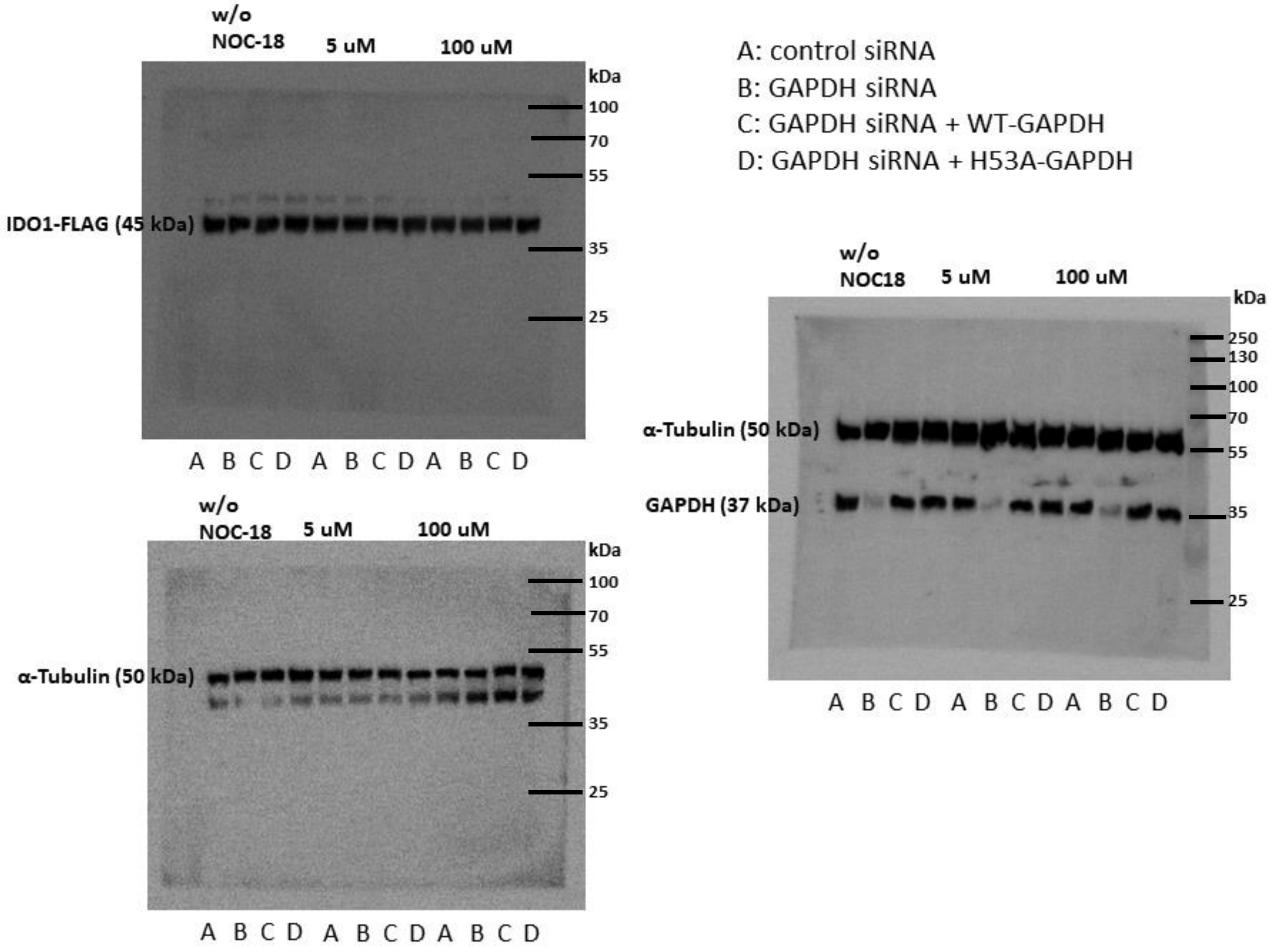

Fig S11: TDO-FLAG expression in GlyA-CHO cells upon silencing of GAPDH with siRNA and complemented with either WT or H53A HA-GAPDH expression from transfected plasmid. GAPDH was effectively silenced as seen in the blot. WB corresponds to Fig. 6.

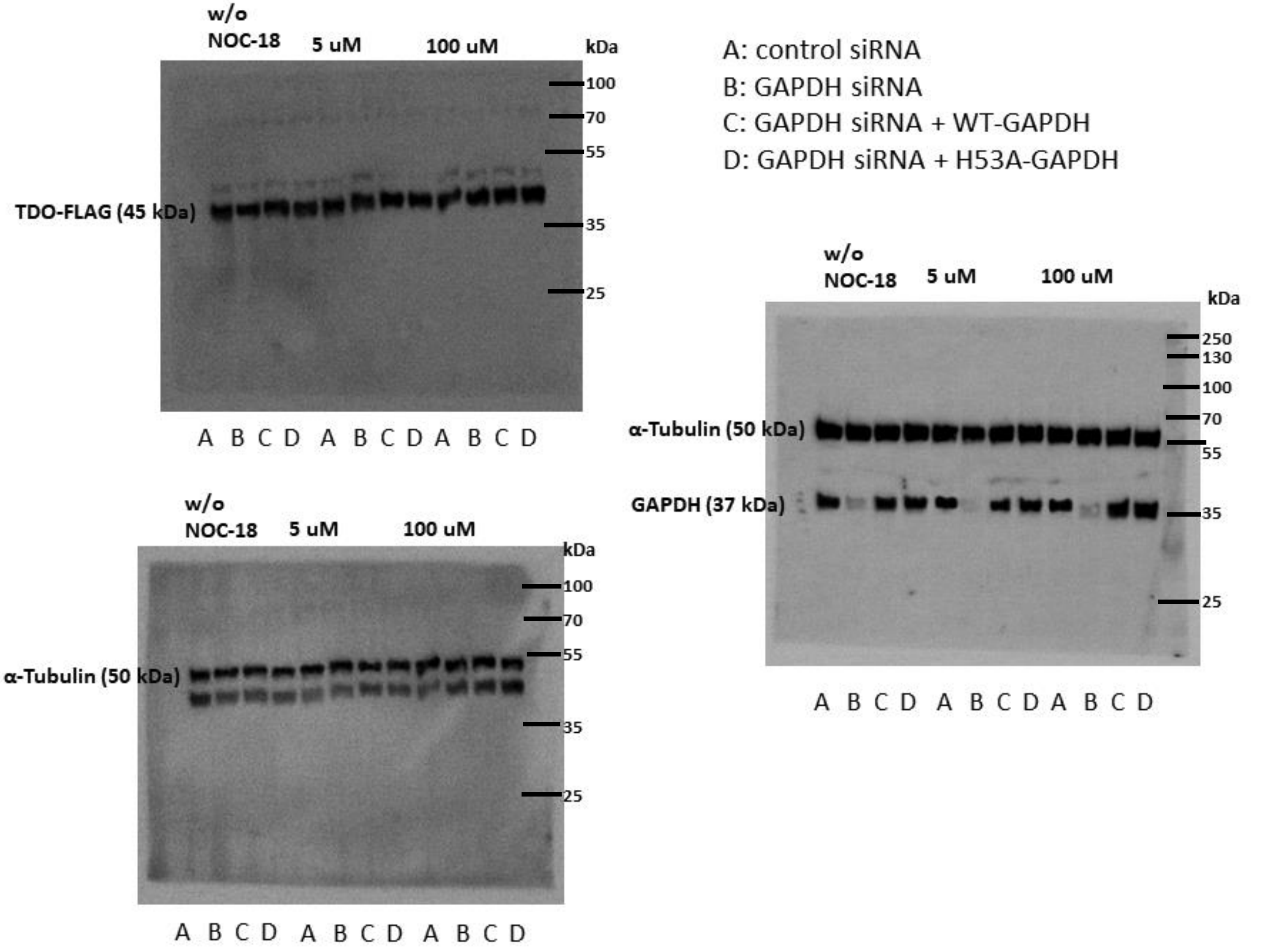

Fig. S12. IDO1-FLAG expression in GlyA-CHO cells upon pre-treatment with 10 µM Radicicol for 6h followed by treatment with different doses of NOC-18 for the indicated time points in presence of Radicicol. WB corresponds to Fig. 7.

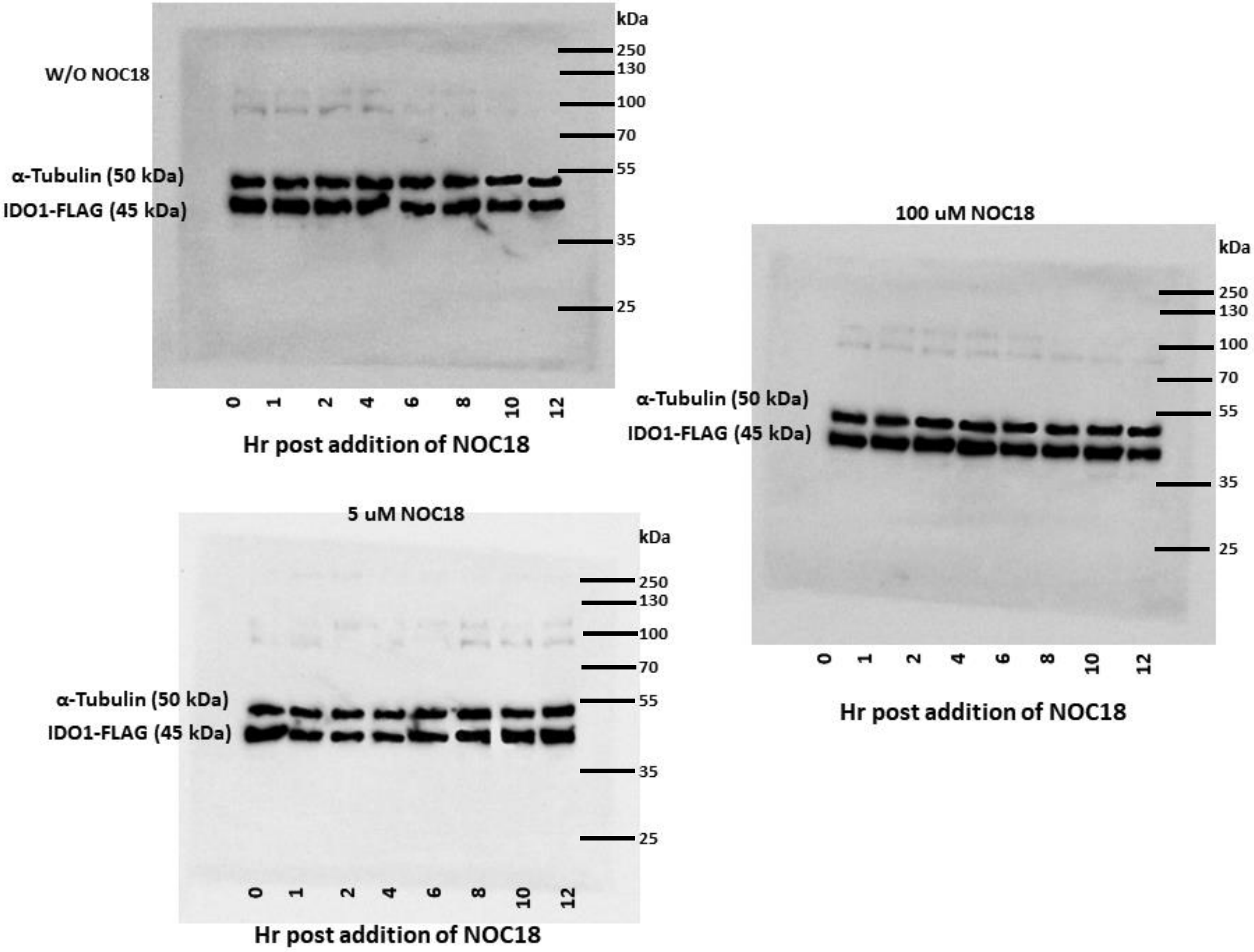

Fig. S13. TDO-FLAG expression in GlyA-CHO cells upon pre-treatment with 10 μM Radicicol for 6h followed by treatment with different doses of NOC-18 for the indicated time points in presence of Radicicol. WB corresponds to Fig. 7.

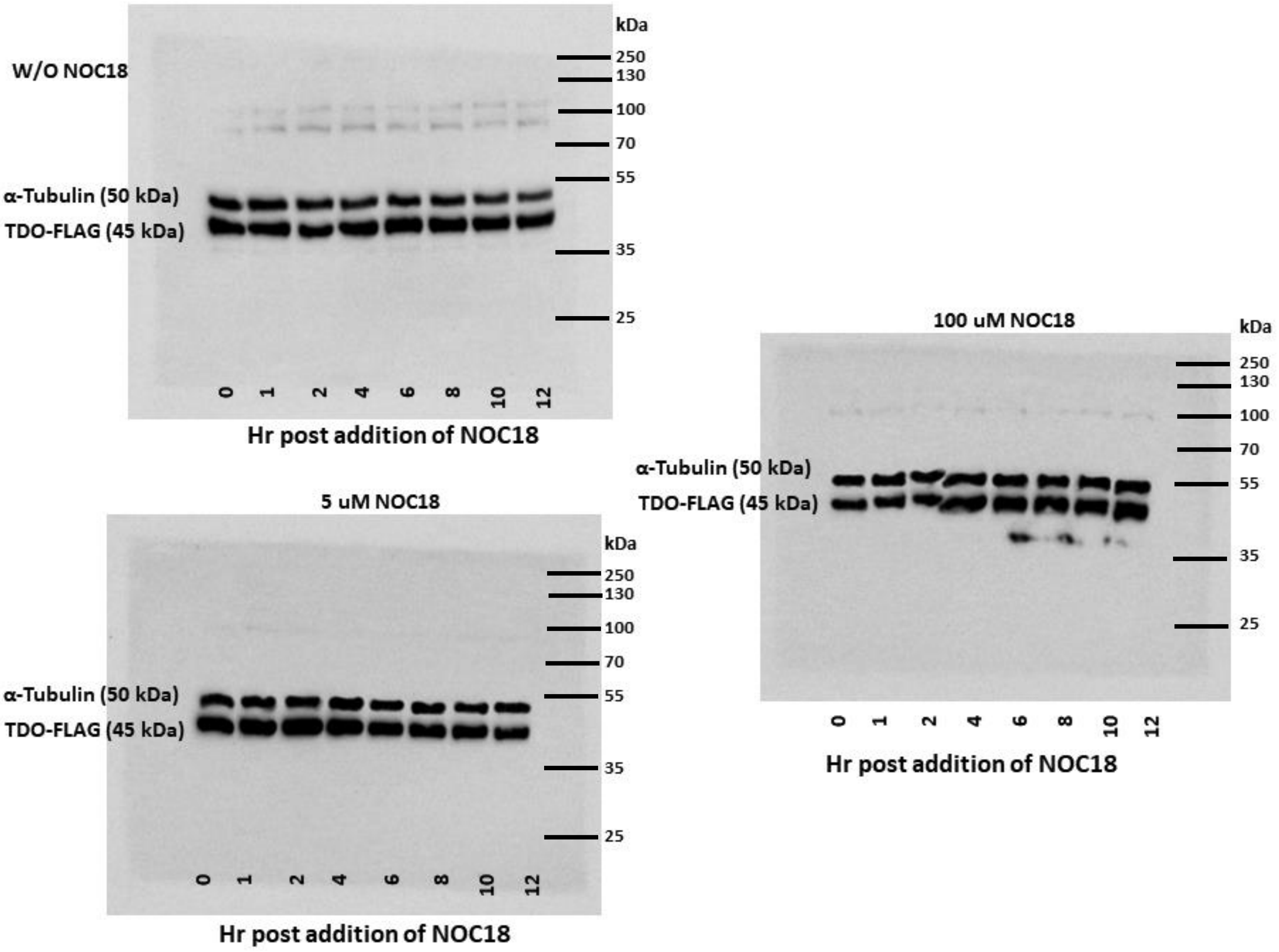

Fig. S14. Background ^{14}C counts due to glycine incorporated into (A) IDO1-FLAG and (B) TDO-FLAG proteins across different time points and different NOC-18 treatment conditions.

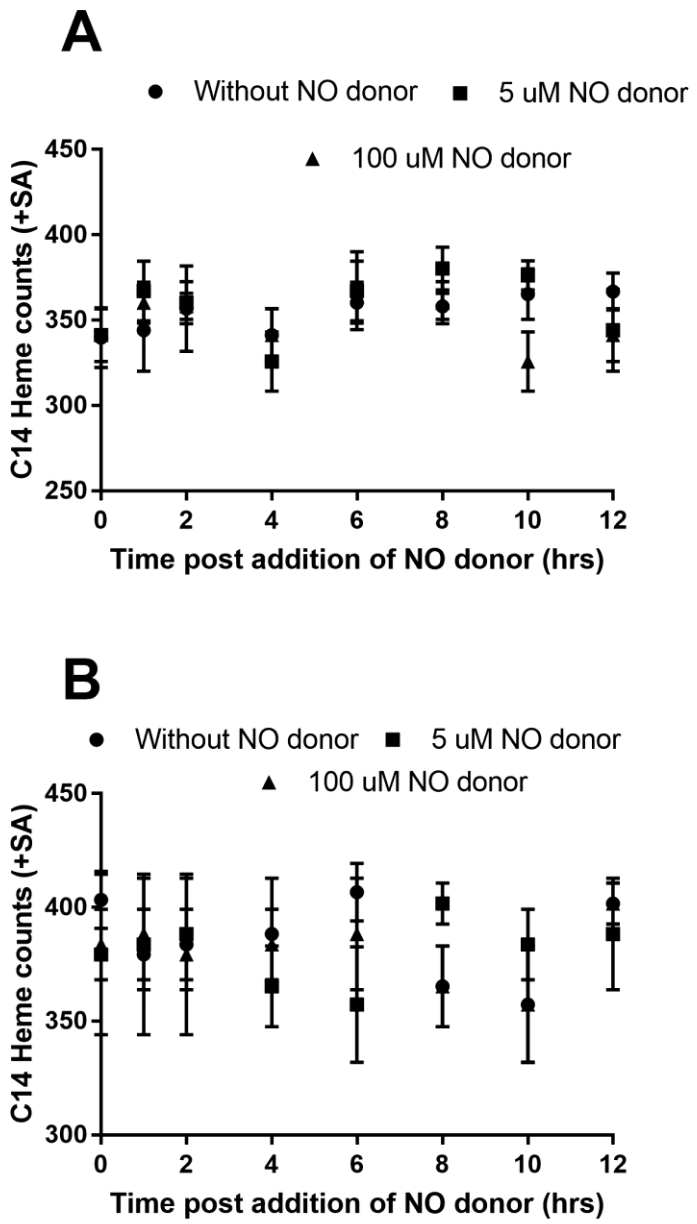

Supplement: Supporting Table S1 and Figures S1–S14 [file mmc1.pdf]
